# Chronic kidney disease recognition amongst physicians and advanced practice providers

**DOI:** 10.1080/0886022X.2021.1974474

**Published:** 2021-09-09

**Authors:** Carlos R. Franco Palacios, Rudiona Hoxhaj, Pankaj Goyal

**Affiliations:** a Nephrology, Critical Care Medicine Department, WellStar Health System, Marietta, GA, USA; b Internal Medicine, WellStar Health System, Marietta, GA, USA; c Division of Nephrology, Kidney C.A.R.E (Clinical Advancement, Research, and Education) Program, University of Cincinnati, Cincinnati, OH, USA

**Keywords:** Chronic kidney disease, diagnosis, provider, renal failure

## Abstract

**Objective:**

Chronic kidney disease is a worldwide public health issue, with increasing prevalence resulting in high morbidity and mortality. As a result, recognizing and treating it early can lead to improved outcomes. We hypothesized that some providers might be more comfortable making this diagnosis than others.

**Methods:**

Retrospective study of 380 patients with chronic kidney disease seen between 2012 and 2016 in an outpatient setting.

**Results:**

Three hundred and sixteen patients were treated by physicians and sixty-four by advanced practice providers. Chronic kidney disease was identified by the primary care providers in 318 patients (83.6%). Patients recognized with chronic kidney disease were older, 76 ± 8.8 vs 72 ± 7.45 years, *p* = 0.001; had lower GFR, 37 [29, 46] vs 57 [37, 76] ml/min/1.73 m^2^, *p* < 0.0001 and were more likely to be seen by a physician compared to an advanced practice provider: 272/316 (86%) vs 46/64 (71.8%), *p* = 0.008. In multivariate analyses, care by a physician, OR = 2.27 (1.13–4.58), *p* = 0.02 was associated with increased recognition of chronic kidney disease. On the other hand, higher GFR was associated with decreased diagnosis of chronic kidney disease, OR = 0.95 (0.93–0.96), *p* < 0.0001.

**Conclusion:**

The odds of chronic kidney disease recognition were higher amongst physicians in comparison to non-physician providers.

## Introduction

Recent assessments of patients in the United States with estimated glomerular filtration rates (GFR) <60 mL/min/1.73 m^2^ showed that only 12% to 20% carried a formal diagnosis of chronic kidney disease (CKD). These patients tend to suffer from poorly controlled hypertension (HTN) and diabetes (DM) with low rates of statins, angiotensin converting enzyme inhibitors (ACEIs) and angiotensin II receptor blockers (ARB) use despite their high cardiovascular risk. [1]

Higher GFR, younger age, female sex, use of serum creatinine without adjusting for GFR and absence of hypertension or diabetes were previously identified as risk factors for missing the diagnosis of CKD in the outpatient setting. [2–4]

With the introduction of GFR, the recognition of CKD, proteinuria testing, prescription of ACEIs/ARBs and avoidance of nephrotoxins has modestly improved. This has led to an increase in Nephrology referrals, especially at earlier stages of CKD. [5–10]

Although there are plenty of studies describing the patient characteristics associated with under-recognition of renal disease, there is paucity of data studying the association of primary care provider characteristics with recognition of CKD. Identifying these factors when present can further increase the quality of care for these patients.

We hypothesized that recognition of CKD can be influenced by factors independent of patient characteristics and that some primary care providers might be more comfortable making the diagnosis of CKD than others.

## Material and Methods

This research was approved by the Institutional Review Board at Rice Memorial Hospital in Willmar Minnesota, protocol 10222014. The study was performed in accordance with the ethical standards as laid down in the 1964 Declaration of Helsinki and its later amendments or comparable ethical standards. Three hundred and eighty patients with CKD seen between 2012 and 2016 in the outpatient setting were included.

Initially, a computer-generated list of patients with GFR less than 60 mL/min/1.73 m^2^ or proteinuria was obtained. The diagnosis of CKD was confirmed by manual revision of the charts.

Chronic kidney disease was defined as evidence of structural or functional kidney abnormalities that persisted for at least 3 months, with or without a GFR of less than 60 mL/min/1.73 m^2^. Proteinuria was defined as urine protein to creatinine ratio ≥200 mg/g per day or urinary albumin excretion ≥300 mg/24 h.

Two types of primary care providers were identified: physicians (MDs or DOs in the specialties of internal medicine and family medicine) and advanced practice providers (nurse practitioners or physician assistants). Each group of providers was solely responsible for delivering primary care to their patients. Each provider’s visit note over a period of 4 years in the medical chart was reviewed, looking for a documented diagnosis of chronic kidney disease or a corresponding ICD-9 or ICD-10 code.

The ICD-9 or ICD-10 codes for proteinuria, chronic kidney disease, chronic renal insufficiency, chronic kidney insufficiency, kidney disease, renal disease, renal insufficiency, kidney stone, renal calculus, renal atrophy, renal sclerosis, kidney failure, renal failure, cystic kidney disease, kidney cyst, polycystic kidney, multicystic kidney, glomerulonephritis, nephritis, nephropathy, and hypertensive chronic kidney disease were used.

### Exclusion criteria

Patients on renal replacement therapy or with a renal transplant; patients younger than 18 years of age or pregnant.

For parametric data, differences in the mean were compared by the Student’s *t*-test. For highly skewed data, the Wilcoxon–Mann–Whitney test was used.

Differences in proportions were assessed by the Chi- square or Fisher’s exact test.

For multivariate analyses, logistic regression models were used for variables with P values < 0.1 in univariate analyses.

P values ≤ 0.05 were considered statistically significant. All the analyses were performed using JMP statistical software version 14 (SAS Campus Drive, Cary, NC).

## Results

Three hundred and sixteen patients were cared by physicians and sixty-four by advanced practice providers. The baseline patient characteristics are described in Table 1 and were similar amongst the providers.

**Table 1.** Patient characteristics by different type of primary care providers.

	Physician providers *N* = 316	Advanced practice providers *N* = 64	*p* Value
Age ± SD, years	75.6 ± 8.53	74.7 ± 9.34	0.45
Male Sex, N (%)	129 (47.1)	30 (46.8)	0.96
Congestive Heart Failure, N (%)	85 (26.9)	12 (18.7)	0.20
Hypertension, N (%)	295 (93.3)	62 (96.8)	0.39
Hyperlipidemia, N (%)	234 (74)	42 (65.5)	0.17
Diabetes mellitus, N (%)	160 (50.6)	33 (51.5)	0.89
Obesity, N (%)	175 (55.3)	36 (56.2)	0.89
Cancer, N (%)	83 (26.3)	13 (20.3)	0.30
Cerebrovascular disease, N (%)	25 (7.91)	6 (9.38)	0.62
Peripheral vascular disease, N (%)	77 (24.4)	19 (29.7)	0.37
Coronary artery disease, N (%)	103 (32.6)	20 (31.2)	0.83
Atrial fibrillation, N (%)	84 (26.6)	11 (17.2)	0.10
Chronic pulmonary disease, N (%)	90 (28.5)	16 (25)	0.56
Chronic liver disease, N (%)	14 (4.43)	2 (3.13)	0.62
Glomerular filtration rate ml/min/1.73 m^2^, median [IQR]	38 [29, 49]	38 [32, 49.7]	0.29
Urinary protein (mg/g), median [IQR]	100 [16, 300]	100 [33, 200]	0.75
Years of practice, median [IQR]	13 [8, 19]	4 [2, 9]	0.007

Abbreviations: SD: standard deviation; Obesity: BMI higher than 30; BSA: body surface area; IQR: interquartile range; Proteinuria: milligram/gram creatinine.

Chronic kidney disease was identified in 318 patients (83.6%) and urinary protein excretion was measured in 321 patients (84.5%).

In patients with proteinuria and GFR below 60 mL/min/1.73 m^2^, seven (25.9%) were not recognized as having CKD. In those with GFR above 60 mL/min/1.73 m^2^, five patients (20%) were not diagnosed with CKD.

One hundred and forty-three patients (45.1%) with recognized CKD were treated with ACEIs vs 33 patients (51.6%) with unrecognized CKD, *p* = 0.40.

Angiotensin receptor blockers were prescribed in 63 patients (19.8%) with CKD diagnosed vs seven patients (11.2%) with undiagnosed CKD, *p* = 0.15.

Non-steroidal anti-inflammatory drugs (NSAIDS) were prescribed in 19 patients (30.5%) not diagnosed with CKD vs 49 patients (15.4%) with CKD diagnosis, *p* = 0.006.

In univariate analyses, the characteristics associated with increased recognition of CKD included older age, lower GFR and care by a physician (Table 2).

**Table 2.** Baseline characteristics associated with recognition of chronic kidney disease.

	Chronic kidney disease recognized *N* = 318	Chronic kidney disease not recognized *N* = 62	*p* Value
Age ± SD, years	76 ± 8.8	72 ± 7.45	0.001
Male gender, N (%)	152 (47.6)	28 (45.1)	0.78
Physician provider, N (%)	272 (86)	44 (14)	0.008
Advanced practice provider, N (%)	46 (71.8)	18 (28.2)	
Years in practice, years, median [IQR]	11 [6, 17]	12.5 [3.25, 17.7]	0.89
Diabetes mellitus, N (%)	161 (50.8)	31 (50)	0.90
Hypertension, N (%)	296 (93.4)	60 (96.8)	0.39
Hyperlipidemia, N (%)	232 (73.2)	43 (69.3)	0.54
Obesity, N (%)	172 (54.3)	38 (61.3)	0.30
Cancer, N (%)	82 (25.9)	14 (22.6)	0.58
Congestive Heart Failure, N (%)	86 (27.1)	11 (17.7)	0.15
Cerebrovascular disease, N (%)	26 (8.20)	5 (8.06)	1
Peripheral vascular disease, N (%)	80 (25.2)	16 (25.8)	1
Atrial fibrillation, N (%)	80 (25.2)	14 (22.6)	0.75
Coronary artery disease, N (%)	102 (32.2)	21 (33.9)	0.88
Pulmonary disease, N (%)	89 (28)	17 (27.4)	1
Chronic liver disease, N (%)	13 (4.10)	3 (4.84)	0.73
Glomerular filtration rate ml/min/1.73 m^2^, median [IQR]	37 [29, 46]	57 [37, 76]	<0.0001
Proteinuria (mg/g), median [IQR]	100 [24.3, 300]	72.6 [14.2, 200]	0.13

In multivariate analyses, lower GFR and care by a physician were independently associated with increased CKD recognition (Table 3).

**Table 3.** Multivariate logistic regression analyses of factors associated with diagnosis of chronic kidney disease.

	OR (95% CI)	*p* Value
Age, per each year change	1.01 (0.98–1.05)	0.30
Glomerular filtration rate per 1 ml/min/1.73 m^2^ increase	0.95 (0.93–0.96)	<0.0001
Physician provider	2.27 (1.13–4.58)	0.02

## Discussion

Chronic kidney disease is a worldwide public health issue, with increasing prevalence and high morbidity and mortality rates. As a result, recognizing CKD and treating it early can lead to improved outcomes.

In the present study, the rate of CKD recognition was high, probably due to the fact that our population was older with a lower baseline GFR, with significant comorbidities and with the GFR automatically included in the electronic medical records. Proteinuria, as recommended in the current guidelines was measured in most patients, likely due to the high prevalence of HTN and DM.

These findings are in line with prior studies which demonstrated that assessing renal function with GFR (versus creatinine alone) translates into increased CKD diagnosis. [11, 12]

Stage 3 CKD (GFR between 30 and 60 mL/min/1.73 m^2^), HTN, proteinuria and DM were prevalent in this population, with increased risks of end- stage renal disease and cardiovascular events. Despite this, 15%–30% of the patients were not recognized as having CKD. Furthermore, 20%–25% of patients with proteinuria were not diagnosed with CKD.

As previously described, unrecognized CKD patients were younger and had a higher GFR, which could have impaired the diagnosis. A significant number of them were not prescribed ACEIs or ARBs and were more likely to be prescribed nephrotoxins, such as NSAIDs.

We found the odds of being diagnosed with CKD were two times higher if the patient was seen by a physician compared to a non-physician provider, despite similar patient characteristics.

One possible explanation for our findings is that physicians had significantly more years of training and practice than advanced practice providers, this could have led to better clinical documentation and pathophysiological reasoning with increased disease recognition. [13, 14]

Since primary care providers are of critical importance in the delivery of medical care, measures to increase the recognition and management of CKD by these practitioners are warranted.

These include access to educational tools, use of electronic clinical decision support systems with treatment recommendations based on the level of renal function and co-management with other professionals, such as nephrologists and pharmacists. These measures have shown to improve the quality of care in CKD (i.e., proper use of ACEIs, ARBs, statins and avoidance of NSAIDs). [15–17]

Similarly, the importance of following GFR trends and the recognition of proteinuria as a marker of adverse renal and cardiovascular outcomes needs to be disseminated among primary care providers.

The above actions could lead to increased testing and diagnosis of CKD, better blood pressure control, timely referral to Nephrology and better renal outcomes. [18–21]

To the best of our knowledge this is the first study assessing the primary care provider characteristics associated with CKD recognition. Limitations of this study include being a retrospective, single center study in a rural setting with a relatively small number of patients. Other shortcomings include the lack of histology and ultrasonography assessment, along with reliance on ICD-9 or ICD-10 codes for diagnosis of some cases of CKD. At the same time, we did not compare treatment decisions or outcomes of care among different providers or specialties.

## Conclusion

In conclusion, we have found that in this high-risk population, the odds of CKD being recognized were higher with physicians in comparison to non-physician providers

## Data Availability

Data is available in a database from the corresponding author on reasonable request.
